# Case Report: Acute kidney injury from chromium exposure—clinical insights

**DOI:** 10.3389/fmed.2025.1692889

**Published:** 2025-12-16

**Authors:** Linlin Xu, Tianxi Chen, Miaojun Shi, Yi Yang

**Affiliations:** 1 Department of Nephrology, The First People's Hospital of Yongkang, Yongkang, China; 2 Department of Nephrology, Center for Regeneration and Aging Medicine, The Fourth Affiliated Hospital of School of Medicine, and International School of Medicine, International Institutes of Medicine, Zhejiang University, Yiwu, China; 3 Zhejiang-Denmark Joint Laboratory of Regeneration and Aging Medicine, Yiwu, China

**Keywords:** acute kidney injury, chromium poisoning, case report, literature review, kidney diseases

## Abstract

This article reports a case of a patient who developed nausea, vomiting, and diarrhea, succeeded by oliguria and acute kidney injury, after exposure to a chromium-containing additive pool water while working. The patient was diagnosed with acute kidney injury, third-degree skin burns, and gastroenteritis. Subsequently, the patient was treated with antioxidant stress management, hemodialysis, and surgical debridement with skin grafting. After 1 year, the patient’s condition gradually improved. In addition, renal biopsy pathology suggested acute interstitial nephritis. In combination, with the medical history and blood chromium monitoring, its kidney pathology was confirmed to be caused by heavy metal chromium poisoning. This case highlights the importance of obtaining a clear history of high exposure to heavy metals for the diagnosis of acute kidney injury, and early active treatment can improve the success rate of rescue.

## 1 Introduction

Chromium is one of the main industrial wastes produced by industries such as textiles, leather manufacturing, electroplating, and metallurgy, posing health problems for humans and animals (1–5). Chromium exists in various oxidation states ranging from −4 to +6, among which Cr(VI) is highly toxic. Cr(VI) is extremely soluble in water and its lethality is more than 100 times that of Cr(III) (6). A number of industrial workers are prone to long-term and extensive exposure to chromium compounds, including: welders in the stainless steel production industry, workers in chromate production and those who work in the electroplating industry. Chronic occupational exposure, environmental pollution, and accidental contact with high concentrations of chromium can cause damage to organs such as the kidneys, liver, skin, gastrointestinal tract and lungs (7–10). Numerous studies (7–12) have shown that Cr(VI) are pathogenic through the induction of oxidative stress, DNA damage, apoptosis and changes in gene expression.

## 2 Case presentation

A 60-year-old male was admitted to the hospital on December 8, 2023, due to “nausea and vomiting for 5 days and diarrhea for 3 days.”

Five days prior to the patient’s current presentation, while working at an electroplating factory, the patient accidentally slipped during the process of cleaning a chemical tank, fell to the ground on the right side of their body, and then got up immediately afterward. Of note, the pool water was a chromium plating solution containing chromium additives(SY-N723) and the patient was not wearing a rubber suit at the time. Three hours later, the patient began to experience symptoms of fatigue, nausea, and discomfort. On the day after the incident, the patient experienced nausea and vomiting, more than 10 times per day, which consisted of gastric contents without coffee-ground material, accompanied by mild abdominal pain, without fever or diarrhea. On day three post-incident, the patient experienced diarrhea, with watery stools 3–4 times per day, accompanied by mild abdominal pain and bloating, reduced urine output, and a sensation of dry mouth. The patient was treated with intravenous fluids (normal saline and pantoprazole injection) at a clinic for 3 days without improvement, and the discomfort gradually worsened. On the afternoon of the fifth day after the incident, the patient was urgently taken to the emergency department of our hospital by family members, and was subsequently transferred to our department for further treatment.

### 2.1 Laboratory tests

On the day of admission, biochemical tests showed: creatinine 1370.2 μmol/L, blood urea nitrogen 42.28 mmol/L, blood glucose 6.36 μmol/L, creatine kinase MB 12.38 ng/mL, lactate dehydrogenase 709 U/L, sodium 129.1 mmol/L, chloride 97.1 mmol/L, calcium 2.01 mmol/L, high-sensitivity troponin 4.77 pg./mL, BNP 474.9 pg./mL. Blood gas analysis: pH 7.202, PCO2 19.1 mmHg, lactate 0.70 mmol/L, osmolality 254.6 mmol/kg. Transaminases were within normal limits.

Physical examination found a blood pressure of 187/92 mmHg and a pulse of 74 beats per minute. The patient was lethargic and their skin presented without jaundice, and petechiae or ecchymosis. There was no coarse breathing and the heart rhythm was regular heart rhythm. On the posterior upper thigh, the wound measured approximately 5.0*10.0 cm, and on the lower thigh, this measured approximately 1.5*2.5 cm. Eschar-like necrosis was noted at the wound base, accompanied by cutaneous discoloration (blackening), loss of tactile pain, and partial subcutaneous fat necrosis.

#### 2.1.1 Diagnosis considered

Acute kidney injury, Third-degree skin burn (TBSA 1%, total body surface area), Gastroenteritis.

#### 2.1.2 Treatment course

On the day of admission, the patient had a 24-h urine output of 50 mL. Therefore, antioxidants including vitamin C, glutathione, sodium thiosulfate, and alpha-lipoic acid were administered [specific dosages as follows: glutathione 1.2 g IV daily (12.18–1.04), vitamin C 2.0 g IV daily (12.18–1.04), alpha-lipoic acid 0.6 g IV daily (12.22–1.04), and sodium thiosulfate 0.64 g IV twice daily (12.28–1.1)]. Hemodialysis was also initiated, with a total of 8 sessions of hemodialysis, 2 sessions of perfusion, and 1 session of hemofiltration performed between December 18 and January 4. On December 23, escharotomy of the right thigh with negative pressure wound therapy (NPWT) for expansion was performed. On December 25, urine output increased to 530 mL, with blood test results showed creatinine at 655 μmol/L and urea nitrogen at 14.07 mmol/L. By December 29, urine output further increased to 1800 mL, and abdominal distension had improved. To determine the cause of acute kidney injury, a renal biopsy was performed on January 1. On January 2, blood biochemical tests revealed a creatinine level of 334 μmol/L and a blood urea nitrogen (BUN) level of 11.6 mmol/L; urinalysis showed weakly positive proteinuria and hematuria. On January 4, the patient’s 24-h urine output reached 3,400 mL, indicating the start of the polyuric phase. On January 5, blood biochemical tests showed a creatinine level of 186 μmol/L and urea nitrogen at 7.48 mmol/L. On the same day, the patient underwent skin debridement and grafting. On January 14, blood showed creatinine at 155 μmol/L and urea nitrogen at 10.39 mmol/L. The patient was discharged on January 15 following clinical improvement. At the outpatient follow-up visit on January 26, the patient had a creatinine level of 101 μmol/L, with successful skin graft survival and good growth. At the 1-year follow-up, the skin damage had healed well.

#### 2.1.3 Pathological examination

Eight glomeruli were observed, with one showing global sclerosis and the others exhibiting mild changes. The capillary loops were well-opened with occasional mild mesangial matrix proliferation. No segmental sclerosis or capsular adhesion was noted, and there were no crescents or necrosis observed. Periodic Acid-Schiff-Methenamine (PASM) staining showed no spike, double track or chain-like structures in the glomerular basement membrane. Some parietal and visceral epithelial cells were found to be swollen, with segmental thickening and partial layering of the capsular wall. Some renal tubular epithelial cells showed granular and vacuolar degeneration, with occasional protein casts and epithelial cell sheddings in the lumen. A loss of brush borders was observed in some renal tubules, with flattened epithelial cells and occasional epithelial cell shedding, which exposed the basement membrane, in addition to occasional mild tubulitis. The interstitium showed diffuse (approximately 75%) edema with lymphocyte/monocyte infiltration, occasional eosinophils/neutrophils; additionally, most tubules in this area were mildly atrophic with minimal fibrosis. No significant changes were observed in the small arteries. IgA, IgG, IgM, C1q, C3, Fib, Kappa, Lambda, and C4 markers were all found to be negative, indicating acute interstitial nephritis.

## 3 Discussion

**Figure 1:**
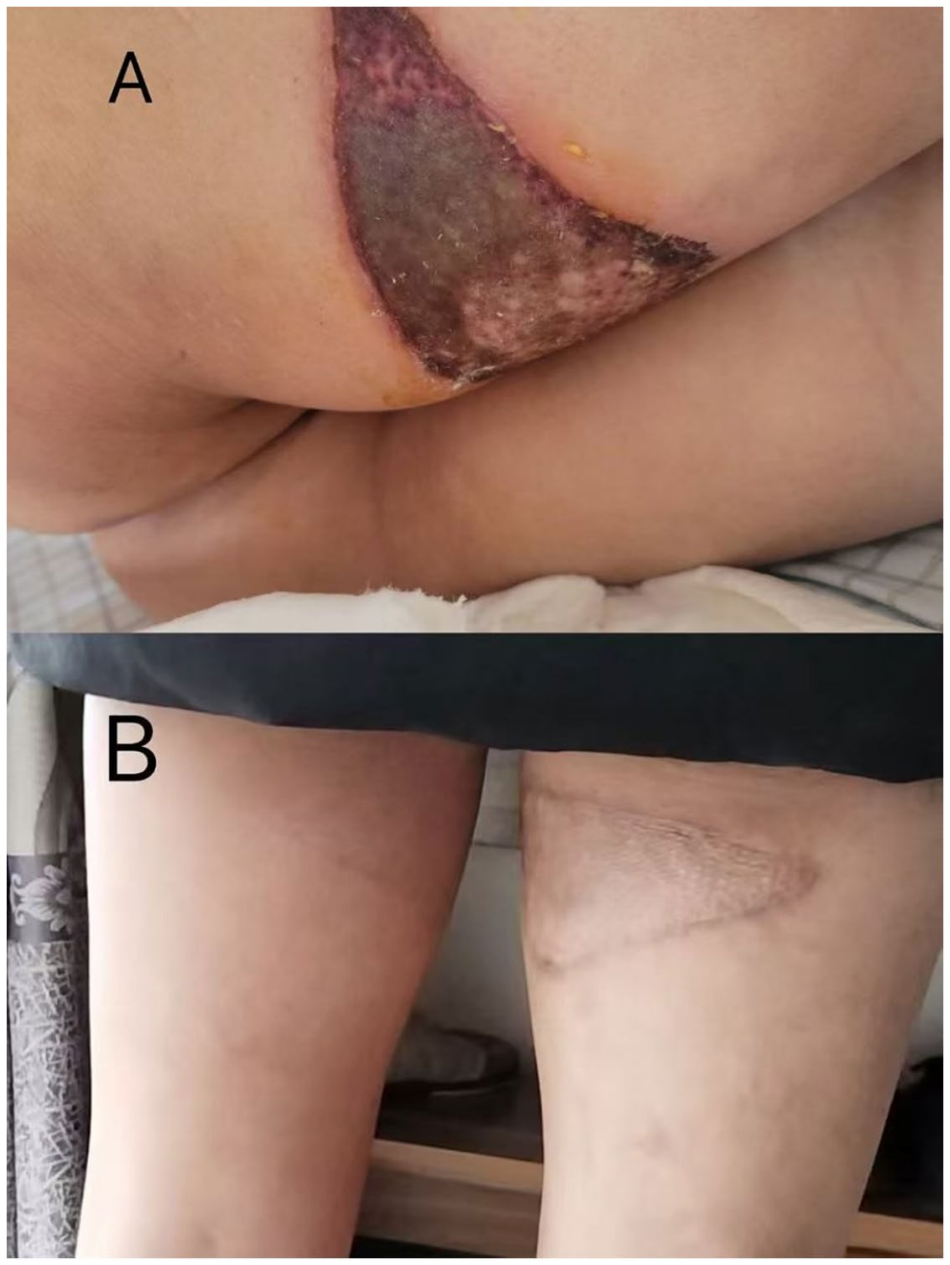
**(A)** The skin condition 5 days after exposure to metallic chromium. **(B)** The condition 1 year after the operation.

**Figure 2:**
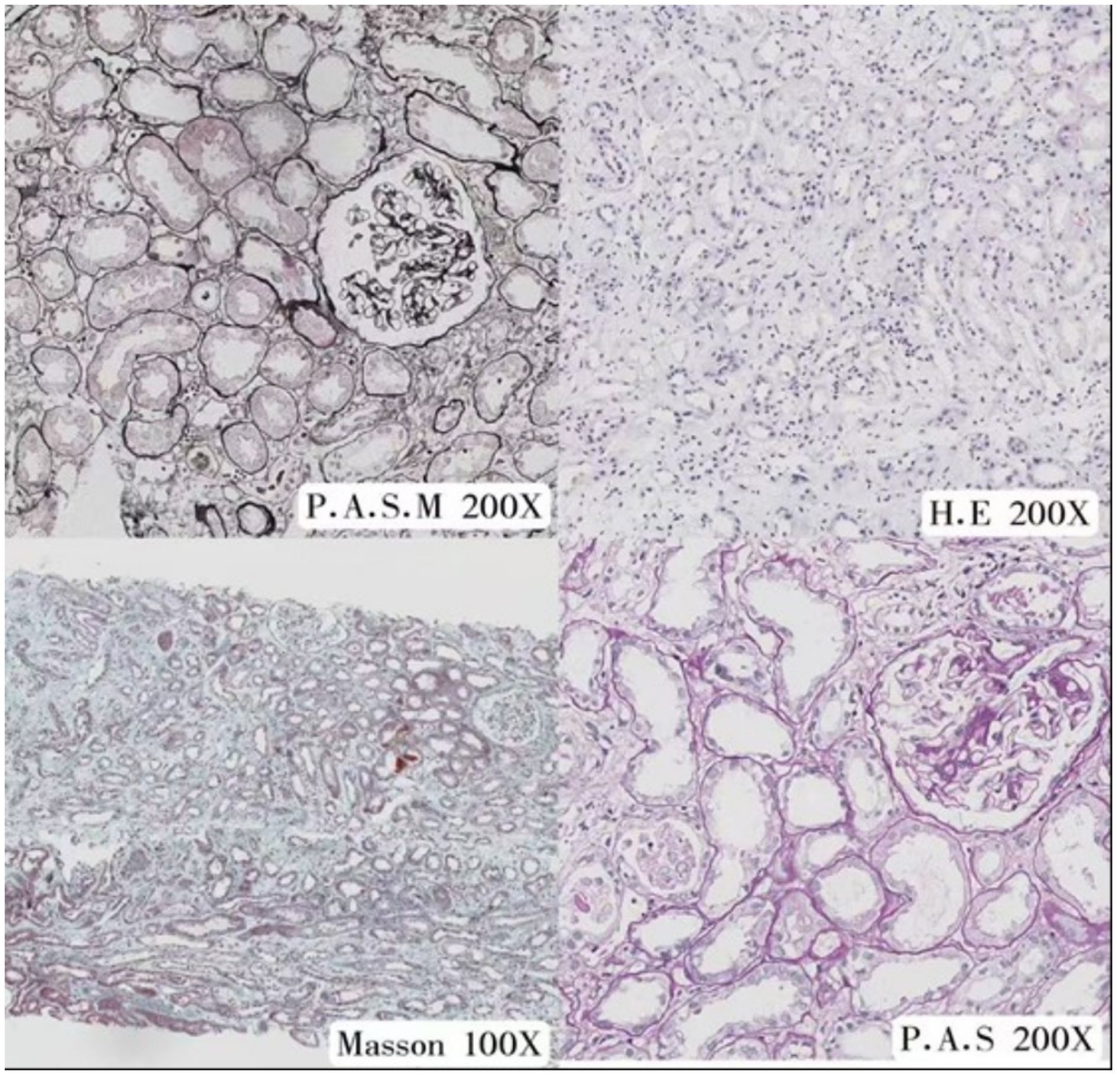
Microscopic images of kidney tissue stained with different techniques: P.A.S.M (Periodic Acid-Schiff-Methenamine Silver) at 200x magnification, H.E (Hematoxylin-Eosin) at 200x, Masson (Masson’s Trichrome Stain) at 100x and P.A.S (Periodic Acid-Schiff) at 200x.

Chromium poisoning can manifest as both acute and chronic conditions (8, 13–16). High-dose chromium exposure can cause acute and severe complications, such as acute renal failure, skin ulcers, gastrointestinal reactions such as nausea and vomiting, and respiratory distress. On the other hand, low-dose chromium exposure poses a hidden threat, necessitating regular monitoring of blood and urine chromium levels, in addition to diagnosing specific complications to facilitate clinical decision making. The toxicity of heavy metals is dose-dependent and time-dependent; once absorbed by the human body, heavy metals tend to be retained and accumulated. Numerous studies have demonstrated that that acute kidney injury caused by chromium is characterized by acute tubular necrosis, whereas chronic exposure often results in tubular atrophy and interstitial fibrosis (16, 17). Generally, the treatment for chromium poisoning involves actively removing chromium from the serum and using antioxidant chelation (18–21) (see Figures 1, 2).

**Figure 3:**
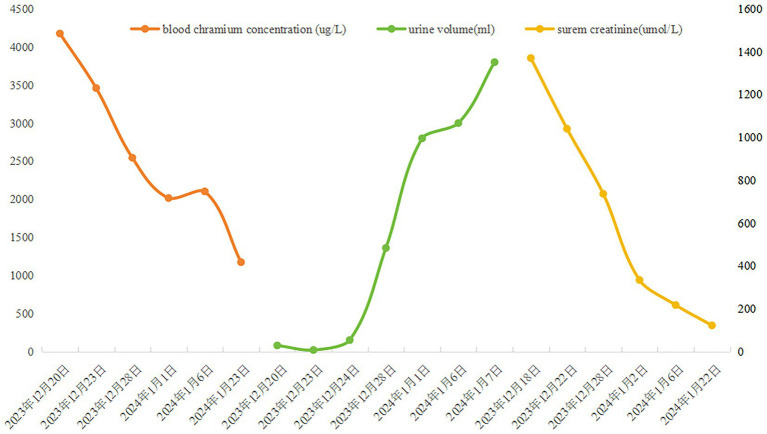
Line graph showing three metrics over time from December 20, 2023, to February 22, 2024. Each metric is represented by different colored lines: orange, green, and yellow, respectively.

This patient initially presented with acute onset of nausea, vomiting, and oliguria due to acute kidney injury. By tracing the patient’s life trajectory to date and their work activities over the past 5 days, a history of high-dose exposure to metallic chromium exposure was confirmed, which provided a clear direction for clinical management. Monitoring of blood chromium levels revealed that the patient’s blood chromium concentration was thousands of times higher than the normal value (see Figure 3). Renal biopsy pathology strongly supported that the cause of the patient’s kidney injury was heavy metal poisoning. Given that the patient was in critical condition upon admission, with life-threatening acute kidney injury, early and adequate hemodiafiltration was administered for resuscitation. Meanwhile, active management of chromium-induced burn injuries to the skin was performed, along with supportive measures such as antioxidant stress management and nutritional support, aiming to improve the likelihood of a favorable outcome.

During the subsequent one-year follow-up period, the patient’s blood chromium level (7.41 μg/L on June 6, 2024, detected by ICP-MS), serum creatinine (67 μmol/L), and urinalysis (proteinuria: negative) were all within the normal range. The patient also noted that such a minor oversight had unexpectedly resulted in a significant loss. He took this chance to urge everyone: always follow operational standards strictly, and if a problem occurs, it’s crucial to seek medical assistance immediately. Although the skin injury left scars, skin function remained unaffected. Acute kidney injury caused by heavy metals is uncommon in this administrative region, and a clear history of high exposure is a necessary condition for diagnosis.

## Data Availability

The original contributions presented in the study are included in the article/supplementary material, further inquiries can be directed to the corresponding author.
